# Polyostotic Fibrous Dysplasia: A Case Report

**DOI:** 10.7759/cureus.65695

**Published:** 2024-07-29

**Authors:** Arun Aram, Karthik Krishna Ramakrishnan, Evangeline P Christina, Paarthipan Natarajan

**Affiliations:** 1 Department of Radiology, Saveetha Medical College and Hospital, Saveetha Institute of Medical and Technical Sciences, Saveetha University, Chennai, IND

**Keywords:** facial distortion, polyostotic, leontiasis ossea, ground glass matrix, fibrous dysplasia

## Abstract

Polyostotic fibrous dysplasia (PFD) is a scarce noncancerous bone condition characterized by the failure to form mature lamellar bone and arrest in the form of woven bone, resulting in deformities and functional limitations. Extreme forms of craniofacial fibrous dysplasia can lead to leontiasis ossea, an exceptionally uncommon presentation. We report a case of a 32-year-old man displaying facial abnormalities indicative of leontiasis ossea. Through radiographic and histopathological assessments, the diagnosis of PFD was confirmed. Surgical intervention was undertaken to address symptoms and enhance facial appearance. This case underscores the diagnostic and therapeutic complexities associated with PFD featuring leontiasis ossea and underscores the significance of a collaborative medical approach.

## Introduction

Polyostotic fibrous dysplasia (PFD) is a rare skeletal disorder characterized by the failure to form mature lamellar bone and arrest as woven bone across multiple bones, leading to deformities, fractures, and significant functional impairments [1]. Among the various manifestations of PFD, leontiasis ossea, or "lion face" syndrome, is particularly notable. This term describes the dramatic enlargement and distortion of facial bones, resulting in a resemblance to the visage of a lion.

While PFD commonly affects the long bones, craniofacial involvement leading to leontiasis ossea is exceptionally rare [2]. The infrequency of this presentation poses considerable diagnostic challenges and necessitates the development of specialized management strategies. Effective management of PFD with leontiasis ossea requires a thorough understanding of its clinical, radiographic, histopathological, and therapeutic aspects.

In this context, we present a unique case report that meticulously details the clinical course, radiographic findings, histopathological characteristics, and therapeutic interventions associated with PFD accompanied by leontiasis ossea. By examining the complexities of this particular case, our aim is to illuminate the diagnostic challenges and therapeutic considerations involved in managing rare skeletal pathologies such as PFD with craniofacial involvement. Through this case study, we seek to enhance the broader understanding and improve the management of these challenging conditions [3].

## Case presentation

A 12-year-old boy presented to our radiology department with progressive facial asymmetry and enlargement observed over the past few years. He reported intermittent headaches and nasal obstruction associated with these facial changes. Physical examination revealed significant facial distortion, characterized by marked prominence of the forehead, cheeks, and jaw, with no evidence of neurological deficits or visual disturbances.

**Figure 1:**
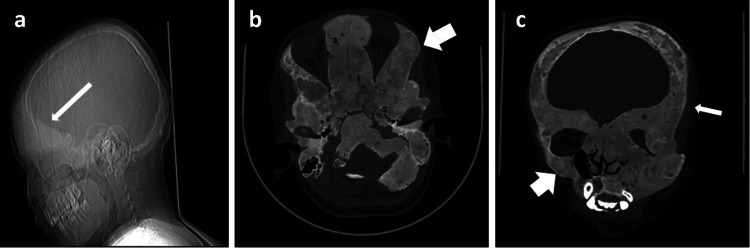
(a) CT topogram (lateral) of a 12-year-old male showing thickened frontal and sphenoid bones (long white arrow); (b) CT plain facial bones (axial section) showing thickened sphenoid bone, clivus, temporal bone, and occipital bones (short white arrow); (c) CT plain facial bones (coronal section) shows diffuse enlargement of the right maxillary (short white arrow) and left frontal bones (long white arrow) and other facial bones with ground glass matrix.

Computed tomography (CT) imaging of the facial bones demonstrated expansion of the diploic space and medullary cavity of calvarial and facial bones, respectively, by ground glass matrix, including the frontal, maxillary, and mandibular bones. There was significant thinning out of the cortical bone, and the medullary cavity became filled with ground glass matrix, particularly in the frontal and maxillary regions (Figure 1).

**Figure 2:**
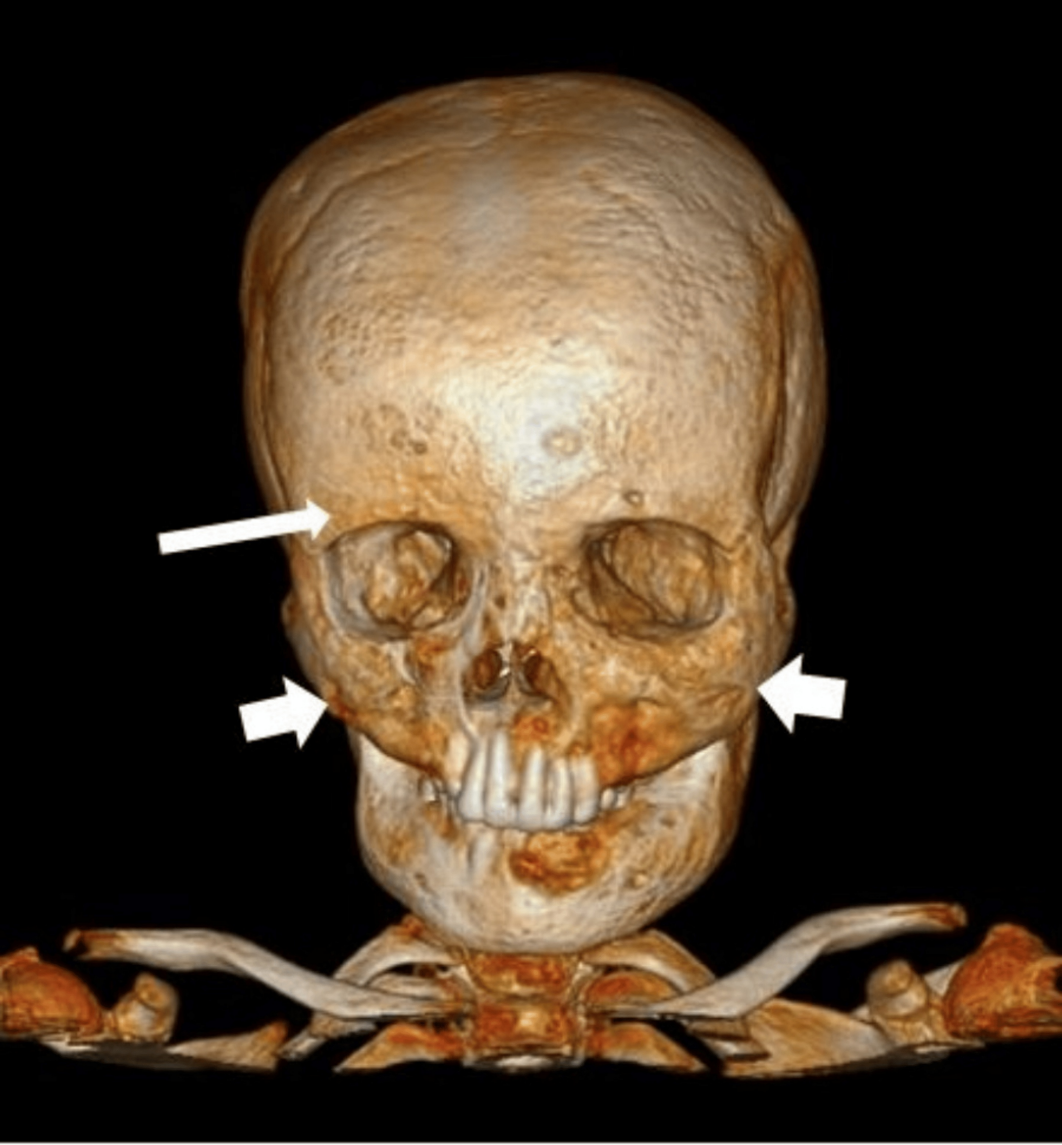
CT 3D reconstruction of facial bones showed diffuse enlargement and coarsening of bilateral frontal (long white arrow) and maxillary bones (short white arrows).

Three-dimensional reconstructions highlighted the extent of bony involvement, revealing significant enlargement and distortion of the facial skeleton, consistent with leontiasis ossea (Figure 2).

**Figure 3:**
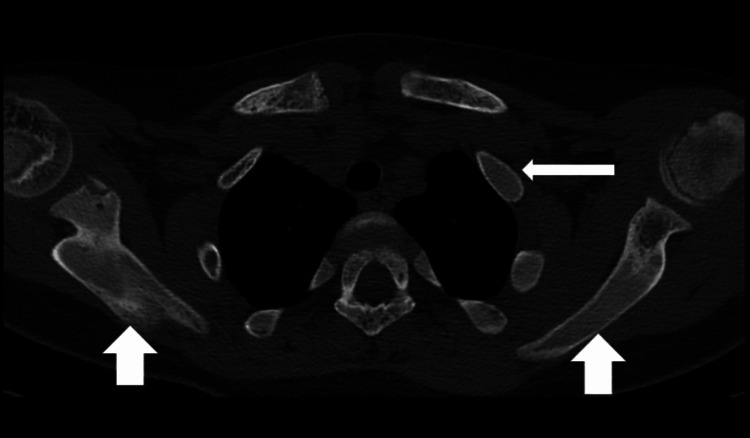
CT plain thorax (axial section) shows diffuse expansion with ground glass matrix involving first, second, and third ribs on the left side (long white arrow) and bilateral scapulae (short white arrow).

Additionally, involvement of the dens of the second cervical vertebra, bilateral first and second ribs, bilateral clavicles, the head of the humerus, and the coracoid and acromion processes of the left scapula was observed. The affected bones exhibited a ground-glass appearance with areas of lucency and sclerosis, which are characteristic features of fibrous dysplasia on CT (Figure 3).

Based on the clinical presentation and radiological findings, a diagnosis of PFD with leontiasis ossea was suspected. A biopsy of the affected bone was performed for histopathological confirmation, which revealed thin, irregular trabeculae of bone within a fibrous stroma and a lack of osteoblastic rimming on the bony trabeculae, consistent with fibrous dysplasia.

The patient's management included conservative measures with nonsteroidal anti-inflammatory drugs (NSAIDs) and bisphosphonate (zoledronate) therapy. No surgical intervention was performed. The patient had a reduction in pain symptoms post-treatment.

## Discussion

PFD is an exceedingly rare skeletal disorder that presents with unique complexity when accompanied by leontiasis ossea, commonly referred to as the "lion face" syndrome. A thorough understanding of its pathogenesis, histopathology, diagnostic methods, imaging findings, and management strategies is crucial for providing effective patient care and addressing the multifaceted challenges associated with this condition [4].

The etiology of PFD is linked to somatic activating mutations of the guanine nucleotide-binding protein (GNAS) gene, resulting in dysregulated activation of the G-protein signaling pathway. This genetic aberration leads to the abnormal proliferation of fibrous tissue within multiple bones, resulting in a spectrum of skeletal abnormalities [5]. Despite this understanding, the precise mechanisms underlying craniofacial involvement and the development of leontiasis ossea remain unclear. Current theories propose that localized somatic mutations during embryonic development trigger dysregulated bone growth patterns, particularly in the craniofacial region.

Histopathological examination of bone biopsy specimens reveals a distinctive pattern characterized by irregularly shaped trabeculae of woven bone embedded within a fibrous stroma [6]. The absence of typical lamellar bone structures and osteoblastic rimming contributes to the ground-glass appearance observed in imaging studies. This histological hallmark aids in confirming the diagnosis and distinguishing PFD from other fibro-osseous lesions.

Diagnosing PFD with leontiasis ossea requires a multifaceted approach. Clinically, patients typically present with striking facial deformities, along with symptoms such as headaches and nasal obstruction [7]. Radiographic imaging, including high-resolution CT scans and zero TE (Time to Echo) magnetic resonance imaging (MRI), provides detailed visualization of bone morphology. These imaging modalities reveal characteristic features such as diffuse sclerosis, cortical thickening, and a ground-glass appearance within the affected bones. Three-dimensional reconstructions further delineate the extent of craniofacial involvement and highlight the distinctive lion-like facial appearance.

Managing PFD with leontiasis ossea requires a coordinated, multidisciplinary effort. Conservative measures, including pain management with nonsteroidal anti-inflammatory drugs (NSAIDs) and bisphosphonate therapy to reduce bone turnover, may offer symptomatic relief and slow disease progression. However, surgical intervention often becomes necessary to address functional impairments and enhance facial aesthetics [8]. Surgical options range from minimally invasive procedures to extensive craniofacial reconstruction, requiring close collaboration between orthopedic surgeons, maxillofacial surgeons, and other specialists.

In summary, PFD with leontiasis ossea represents a rare and intricate clinical entity, emphasizing the importance of a comprehensive understanding of its pathogenesis, histopathology, diagnostic modalities, imaging findings, and management strategies [9]. Through a multidisciplinary approach that integrates clinical expertise, advanced imaging techniques, and histopathological analysis, optimal patient outcomes can be achieved, ultimately improving the quality of life for individuals affected by this challenging skeletal disorder [10].

Review of literature

The previously published literature on PFD has been reviewed and compiled in the form of a table (Table 1).

**Table 1:** Review of literature table FD - fibrous dysplasia; MAS - McCune-Albright syndrome

References	Topics	Key points	Significance
Kushchayeva et al., 2018: Fibrous dysplasia for radiologists: beyond ground glass bone matrix. *Insights Imaging*, 9:1035-1056. 10.1007/s13244-018-0666-6 [1]	Radiological features of fibrous dysplasia (FD)	- FD characterized by ground glass matrix, endosteal scalloping, cortical thinning. - Differentiation between monostotic and polyostotic FD.	Provides comprehensive radiologic criteria for diagnosing and managing FD, aiding in distinguishing it from other pathologies.
Robinson et al., 2016: Fibrous dysplasia/McCune-Albright syndrome: clinical and translational perspectives. *Curr Osteoporos Rep*, 14:178-186. 10.1007/s11914-016-0317-0 [2]	Clinical and translational aspects of FD/McCune-Albright syndrome (MAS)	- Clinical spectrum of FD and MAS. - Advances in understanding the molecular pathogenesis.	Highlights the clinical variability and genetic underpinnings of FD/MAS, important for personalized treatment strategies.
Lee et al., 2002: Normal vision despite narrowing of the optic canal in fibrous dysplasia. *N Engl J Med*, 347:1670-1676. 10.1056/NEJMoa020742 [3]	Impact of FD on optic canal and vision	- FD can cause optic canal narrowing. - Patients can maintain normal vision despite significant narrowing.	Provides evidence that optic nerve decompression may not be necessary in all cases of optic canal involvement in FD.
Chapurlat et al., 2008: Fibrous dysplasia of bone and McCune-Albright syndrome. *Best Pract Res Clin Rheumatol*, 22:55-69. 10.1016/j.berh.2007.11.004 [4]	Comprehensive review of FD and MAS	- Pathophysiology, clinical presentation, and management of FD/MAS. - Discussion on bisphosphonates and surgical options.	Summarizes current knowledge and treatment approaches, serving as a valuable reference for clinicians.
Boyce et al., 1993: Fibrous dysplasia/McCune-Albright syndrome. In Adam MP, Ardinger HH, Pagon RA, et al. (Eds.), University of Washington, Seattle. [5]	Textbook on FD/MAS	- Detailed overview of FD/MAS including genetics, clinical features, and management. - Genetic and molecular insights.	A foundational resource providing a thorough understanding of FD/MAS, used for educational and clinical guidance.
Dalle Carbonare et al., 2022: Surgical management of syndromic versus non-syndromic craniofacial fibrous dysplasia: a systematic review and meta-analysis. *Br J Oral Maxillofac Surg*, 60:1166-1175. 10.1016/j.bjoms.2022.06.002 [6]	Surgical management of craniofacial FD	- Comparison of surgical outcomes in syndromic vs. non-syndromic FD. - Meta-analysis of surgical interventions.	Provides evidence-based guidelines for surgical approaches, highlighting differences in outcomes based on FD subtype.
Chapurlat et al., 2021: Bisphosphonates for the treatment of fibrous dysplasia of bone. *Bone*, 143:115784. [7]	Bisphosphonate therapy in FD	- Evaluation of bisphosphonates in reducing FD-related bone pain and lesions. - Long-term outcomes and side effects.	Assesses the effectiveness and safety of bisphosphonates, informing therapeutic decisions for FD patients.
Kang et al., 2015: A Novel and Easy Approach for Contouring Surgery in Patients With Craniofacial Fibrous Dysplasia. *J Craniofac Surg*, 26:1977-8. 10.1097/SCS.0000000000001953 [8]	Contouring surgery techniques in craniofacial FD	- Introduction of a novel surgical approach for craniofacial contouring. - Practical steps and case outcomes.	Enhances surgical options for craniofacial FD, providing a simpler technique for better aesthetic results.
Ricalde et al., 2001: Craniofacial fibrous dysplasia of the fronto-orbital region: A case series and literature review. *J Oral Maxillofac Surg*, 59:157-167. 10.1053/joms.2001.20487 [9]	Case series on craniofacial FD	- Clinical features and surgical management of fronto-orbital FD. - Review of literature on surgical outcomes.	Offers insights into the complexities of treating fronto-orbital FD and summarizes current practices and outcomes.
Fitzpatrick et al., 2004: Imaging findings of fibrous dysplasia with histopathologic and intraoperative correlation. *AJR Am J Roentgenol*, 182:10.2214/ajr.182.6.1821389 [10]	Imaging and histopathology of FD	- Correlation between imaging findings and histopathology. - Role of imaging in diagnosis and surgical planning.	Improves understanding of the radiological and pathological features of FD, aiding in accurate diagnosis and effective treatment planning.

## Conclusions

The occurrence of PFD with leontiasis ossea represents an exceptionally rare manifestation of this skeletal disorder. Swift diagnosis and appropriate management are imperative to mitigate symptoms, prevent complications, and enhance the patient's quality of life. An integrated approach involving radiologists, orthopedic surgeons, maxillofacial surgeons, and endocrinologists is essential for providing optimal patient care. Further research is warranted to deepen our understanding of the underlying pathophysiology and to explore innovative therapeutic approaches for this uncommon condition.

## References

[1] Kushchayeva YS, Kushchayev SV, Glushko TY, Tella SH, Teytelboym OM, Collins MT, Boyce AM (2018). Fibrous dysplasia for radiologists: beyond ground glass bone matrix. Insights Imaging.

[2] Robinson C, Collins MT, Boyce AM (2016). Fibrous dysplasia/McCune-Albright syndrome: clinical and translational perspectives. Curr Osteoporos Rep.

[3] Lee JS, FitzGibbon E, Butman JA (2002). Normal vision despite narrowing of the optic canal in fibrous dysplasia. N Engl J Med.

[4] Chapurlat RD, Orcel P (2008). Fibrous dysplasia of bone and McCune-Albright syndrome. Best Pract Res Clin Rheumatol.

[5] Szymczuk V, Florenzano P, de Castro LF, Collins MT, Boyce AM (2015). Fibrous dysplasia/McCune-Albright syndrome. GeneReviews®.

[6] Dalle Carbonare M, Manisali M (2022). Surgical management of syndromic versus non-syndromic craniofacial fibrous dysplasia: a systematic review and meta-analysis. Br J Oral Maxillofac Surg.

[7] Chapurlat R, Legrand MA (2021). Bisphosphonates for the treatment of fibrous dysplasia of bone. Bone.

[8] Kang SJ, Oh MJ, Jeon SP (2015). A novel and easy approach for contouring surgery in patients with craniofacial fibrous dysplasia. J Craniofac Surg.

[9] Ricalde P, Horswell BB (2001). Craniofacial fibrous dysplasia of the fronto-orbital region: a case series and literature review. J Oral Maxillofac Surg.

[10] Fitzpatrick KA, Taljanovic MS, Speer DP, Graham AR, Jacobson JA, Barnes GR, Hunter TB (2004). Imaging findings of fibrous dysplasia with histopathologic and intraoperative correlation. AJR Am J Roentgenol.

